# Spared nerve injury rats exhibit thermal hyperalgesia on an automated operant dynamic thermal escape Task

**DOI:** 10.1186/1744-8069-1-18

**Published:** 2005-05-26

**Authors:** Marwan Baliki, Oscar Calvo, Dante R Chialvo, A Vania Apkarian

**Affiliations:** 1 Department of Physiology, Northwestern University Medical School, Chicago, IL, 60611, USA

**Keywords:** Neuropathic Pain, Spared Nerve Injury, Chronic Constriction Injury, Allodynia, Pain Behavior, rat, mouse

## Abstract

Well-established methods are available to measure thermal and mechanical sensitivity in awake behaving rats. However, they require experimenter manipulations and tend to emphasize reflexive behaviors. Here we introduce a new behavioral test, with which we examine thermal sensitivity of rats with neuropathic injury. We contrast thermal hyperalgesia between spared nerve injury and chronic constriction injury rats. This device is a fully automated thermal sensitivity assessment tool designed to emphasize integrated learned responses to thermal painful and non-painful stimuli that are applied dynamically to a surface on which the animal is standing. It documents escape behavior in awake, unrestrained animals to innocuous and noxious heating of the floor where the animal is located. Animals learn to minimize pain by escaping to the opposite non-heated side; escape latency is recorded. On this device, thermal stimulus-response curves showed > 6°C leftward shift in both groups of neuropathic rats. In contrast, when these animals were tested on hotplate the stimulus-response shift was < 2°C. Spared nerve injury rats showed even less evidence for thermal hyperalgesia when thermal sensitivity was tested by measuring paw withdrawal to infrared heating, plantar test. The implications of test dependent magnitude of thermal hyperalgesia are discussed from the viewpoint of the tests used, as well as the animal models studied. It is argued that the dynamic thermal operant task reveals the relevance of the neuropathic injury associated pain-like behavior in relation to the whole organism.

## 1. Introduction

The discovery of animal models that exhibit different elements of clinical pain syndromes, coupled with advances in tools for quantification of pain behavior, have greatly advanced understanding of mechanisms involved in acute and chronic pain. Pain in animals can only be determined by evaluating behavioral cues. Animal studies measure two types of pain behavior: simple withdrawal reflexes and complex voluntary and intentional behaviors [1]. Various methods have been used in assessing reflexive pain behaviors such as tail flick, limb withdrawal, or orofacial reflexes in response to acute painful stimuli. However, reflex behavior as a measure of pain perception has long been debated among pain researchers. Chapman [1] argued that they could be merely a measure of reflex activity instead of true pain sensation since reflexes can be exerted in spinalized or anesthetized animals. For example, the tail flick and paw withdrawal responses can be elicited in spinal animals [2] and therefore represent spinal reflexes; while vocalization [3] and paw licking on the hotplate test can be elicited in decerebrate animals [3-6] and therefore represent spinal-bulbospinal reflexes. Other investigators also suggest that changes in reflex activity might be due to alterations in motor as well as sensory processing [7,8]. Considerably less effort has been dedicated to measure supraspinal nocifensive behaviors that require integrated behavior, which may be more dependent on cortical activity.

The most commonly used stimulus modalities are electrical, mechanical and thermal. The adequacy and shortcomings of each of these modalities has also been widely debated. Electrical stimulation has been criticized since skin receptors are bypassed and synchronous afferent patterns are generated [9]. Although stimulus location and current density can be well controlled with electrical stimulation, it usually requires restraining the animal thus leading to high levels of anxiety and stress that are known to exhibit modulatory effects on pain sensitivity [10-12]. Commonly used mechanical tests utilize the assessment of paw withdrawal latencies and/or observations of guarding behavior to certain mechanical stimuli [13], such as thresholds to withdrawal to von Frey filaments and to pin prick. A present difficulty with mechanical tests is that the characteristics of mechanical nociception (e.g. combinations of diameter and force in the von Frey test) remain unknown and variable across body sites [14]. Thermal stimulation has been extensively used in assessing pain behavior in animals [9] and it remains the basis of most pain assessment tools, like the tail flick test [15], the hind-limb withdrawal plantar test [16] and the hotplate test [17,18]. The advantages of using heat are the relative constancy of its threshold across body sites, and the extensive research done in psychophysical and physiological studies that has defined and established the range of temperatures that produce heat nociception and its underlying mechanisms. Thus, responses to painful thermal stimuli remain one of the best behavioral tools for studying pain in animals.

The most widely used animal pain assessing tools entail manually recording the duration of animals' limb, or other body parts, withdrawal after applying acute noxious or non-noxious stimuli, or to manually measure and record the latency of licking behaviors, most commonly on the hot plate test (see [19] for latest review). Recent studies examined the interaction between the lab environment and measures of pain behaviors in rodents [20,21]. Chesler et al. [20] compared more than 5,000 data points collected from adult mice, where tail withdrawal latencies were measured for a 49 °C stimulus. A major source of the variance was attributed to experimenter-related variability. Given that all commonly used pain behavior measures depend on the interaction between the experimenter and the animal, the experimenter bias and inability to perform the measures in a blinded fashion remain important shortcomings. Therefore, we developed: 1) A fully automated thermal pain behavior assessment tool, 2) to evaluate pain behavior in response to thermal innocuous and noxious stimuli, 3) in awake unrestrained rodents in a task that requires learning, and 4) to produce a more objective measure of pain behavior.

Patrick Wall and colleagues were the first to recognize that injury to a peripheral nerve may generate pathophysiological mechanisms that are different from those elicited by discrete acute noxious stimuli [22]. Since then, many important animal models for acute and chronic pain have been established and proved useful in unraveling their mechanisms. Here we examine pain behavior in two animal models for neuropathic pain, the chronic constrictive injury (CCI) and the spared nerve injury (SNI) models, and compare the results to standard pain assessing tools. The CCI model was developed by Bennett and Xie [23] and has been extensively studied since; it mimics important clinical chronic pain symptoms such as heat hyperalgesia and mechanical allodynia. SNI model was more recently established by Décosterd and Woolf [24], and was shown to robustly express mechanical and cold allodynia. However, in contrast to the CCI model, SNI rats did not show heat hyperalgesia on the plantar test. Here we test both neuropathic models for mechanical, cold and thermal sensitivity. We examine thermal sensitivity using three tests: plantar, hotplate, and our new automated operant dynamic thermal paradigm (AlgoTrack).

## Results

Rats subjected to SNI and CCI showed signs of neuropathy on the operated paw 7 days after induction of injury. The neuropathic behavior included abnormal positioning of the paw (inversion) and signs of spontaneous pain such as shaking, licking and biting of the injured paw. Manifestations of the neuropathic pain were also evident from the behavioral pain tests. Injured rats also displayed trophic changes, which were most evident as abnormal growth of the nails and decreased grooming behavior. The operated animals (SNI, CCI, and sham) did not exhibit any evident motor dysfunctions post surgery.

### Mechanical sensitivity

**Figure 1:**
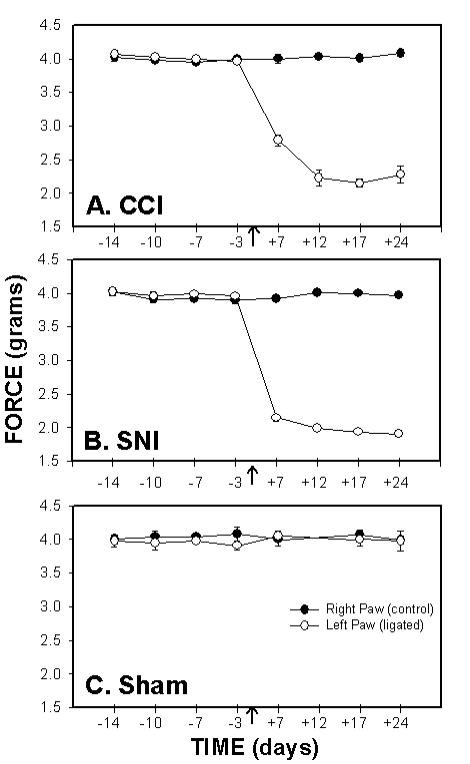
*Mechanical sensitivity*, force (g) required for 50% threshold for paw withdrawal, as a function of time in CCI (A), SNI (B) and sham (C) rats. Mechanical paw-withdrawal thresholds of the ligated paw were significantly attenuated after ligation (indicated by an arrow) in the CCI and SNI models as compared to the right paw (control), and to sham. In both groups, increased mechanical sensitivity persisted for 24 days after ligation.

Mechanical paw-withdrawal thresholds, in grams, of the injured (left) paw decreased significantly in both SNI and CCI animals as compared to the control (right) paw and to sham, 7 days post ligation. This attenuation in mechanical thresholds peaked during the second week following injury and was maintained throughout the period of testing (Fig. 1). 3-way ANOVA for mechanical thresholds as a function of animal grouping, day of test, and paw tested was highly significant for all 3 factors (for animal grouping F_2,199 _= 370.; for test day F_3,199 _= 157.; and for paw tested F_3,199 _= 1407.; p < 10^-5 ^for all 3 factors), for their pair-wise interactions (p < 10^-5^) and for their 3 way interaction (p < 10^-5^).

### Cold sensitivity

**Figure 2:**
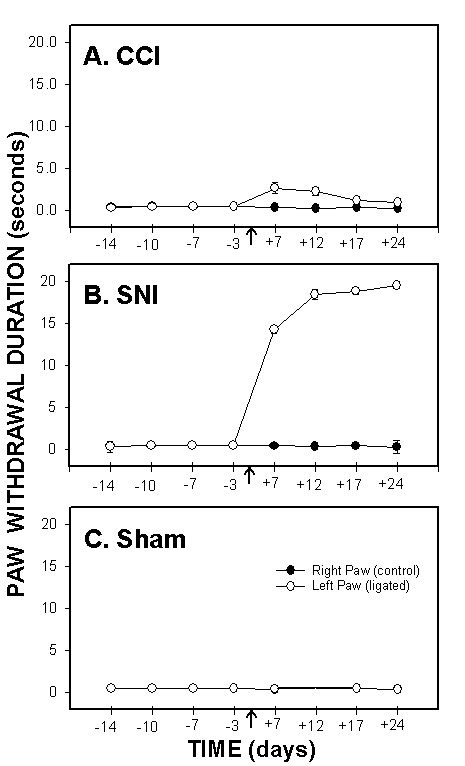
*Cold sensitivity*, paw-withdrawal duration to acetone, as a function of time in CCI (A), SNI (B) and sham (C) rats. Paw withdrawal duration to acetone applied to the ligated paw was significantly increased after ligation (indicated by an arrow) in SNI and CCI animals as compared to the right paw (control) and to sham. Increased cold sensitivity was maintained for 24 days after ligation in both groups. Cold sensitivity change was much smaller in CCI animals than in SNI rats.

Both SNI and CCI rats exhibited significant changes in cold sensitivity after ligation, although the magnitude of change in SNI rats was ten times larger than in CCI rats. Paw-withdrawal duration to cold, as measured by the acetone drop test increased at day 7, peaked at day 12, and was maintained for 24 days following ligation (Fig. 2). 3-way ANOVA for paw-withdrawal duration as a function of animal grouping, day of test, and paw tested was highly significant for all 3 factors (for animal grouping F_2,199 _= 997.; for test day F_3,199 _= 137.; and for paw tested F_1,199 _= 1142.; p < 10^-5 ^for all 3 factors), for their pair-wise interactions (p < 10^-5^) and for their 3 way interaction (p < 10^-5^).

### Plantar test responses

**Figure 3:**
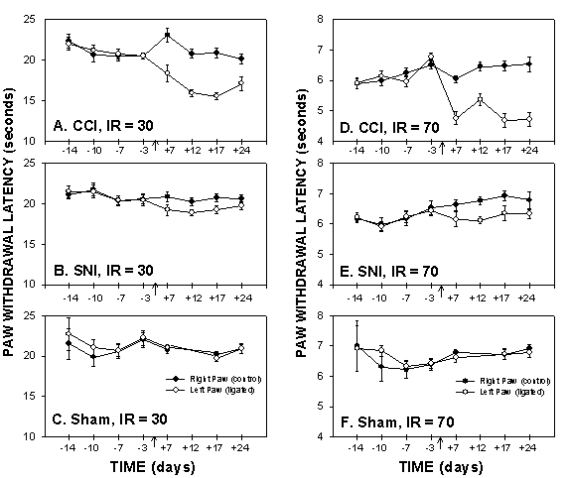
*Plantar test for thermal sensitivity*, paw-withdrawal latencies, as a function of time in CCI, SNI and sham rats, performed at 2 Infra Red (IR) intensities, 30 (A, B, and C) and 70 (D, E, and F). Paw withdrawal latencies of the ligated paw decreased in CCI and SNI rats 7 days post ligation as compared to the control (right) paw and to sham animals. Thermal hyperalgesia, as assessed on plantar test, was smaller in SNI than CCI rats.

Both SNI and CCI rats exhibited significant increases in thermal sensitivity measured by plantar test after ligation, although the magnitude of change in SNI rats was half as large as in CCI rats. Paw-withdrawal latency to IR heat decreased by day 7 and was maintained for 24 days after ligation (Fig. 3). 4-way ANOVA for paw-withdrawal latency as a function of animal grouping, day of test, paw tested, and IR heat intensity (IR = 30, 70) was highly significant for all 4 factors (for animal grouping F_2,396 _= 13.8; for test day F_3,396 _= 6.2.; for paw tested F_1,396 _= 34; and for heat intensity F_1,396 _= 6393.7; p < 10^-5 ^for all 4 factors). All pair-wise interactions were significant except animal grouping with heat intensity. All 3-way interactions were not significant except for the animal group, paw, and heat intensity interaction (F_2,396 _= 3.8; p < 0.02). The 4-way interaction was not significant. Also, the contrast between paws (injured and non-injured) in SNI rats was not significant for higher intensity heat (IR = 70 F_1,396 _= 1.2, p < 0.27) in contrast to lower intensity heat in SNI rats (IR = 30 F_1,396 _= 9.6, p < 0.002) and to CCI (IR = 70 F_1,396 _= 56.6, p < 10^-5^; IR = 30 F_1,396 _= 19.8, p < 0.01).

### Hot plate test responses

**Figure 4:**
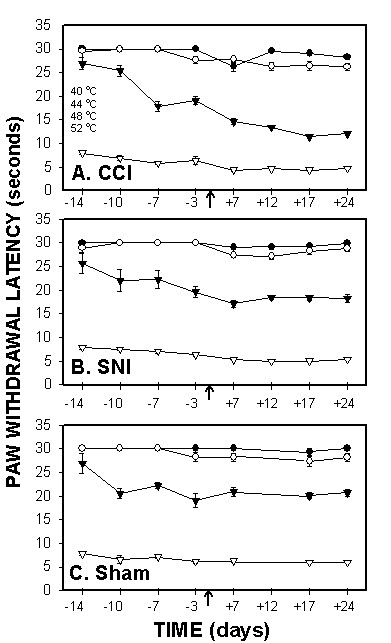
*Hotplate test for thermal sensitivity*, paw withdrawal latencies, as a function of time in CCI (A), SNI (B) and sham (C) rats, performed at 4 temperatures (40, 44, 48 and 52°C). Response latency decreases were most evident in CCI rats when tested at 48°C.

Hot plate test was performed for 4 different temperatures (40, 44, 48, and 52°C). At 40 and 44°C, most animals did not exhibit a positive response within the cut-off limits (30 seconds) pre and post ligation. However, for 48 and 52°C, animals responded within the 30 seconds limit and the response was temperature dependent (Fig. 4). 3-way ANOVA for hind-paw withdrawal latency as a function of animal grouping, day of test, and floor temperature (40, 44, 48, and 52°C) was highly significant for all 3 factors (for animal grouping F_2,392 _= 224.; for test day F_3,392 _= 80.; and for floor temperature F_3,392 _= 2,442.; p < 10^-5 ^for all 3 factors). All pair-wise interactions and the 3-way interaction were significant. Contrasts between pre- and post surgery hind-paw withdrawal latencies were significant for CCI (F_1,392 _= 56.7, p < 10^-5^) and SNI (F_1,392 _= 13.8, p < 0.0002) but not for sham (F_1,392 _= 0.01, p > .9) animals. The largest difference between pre- and post surgery responses were observed at 48°C stimuli in CCI rats (F_1,392 _= 56.7, p < 10^-5^). In SNI rats, pre- vs. post-surgery responses were significantly different only at 44°C (F_1,392 _= 5.2, p < 0.03) and 48°C stimuli (F_1,392 _= 8.6, p < 0.004). The contrasts between CCI and sham, and between SNI and sham were significant across days and temperatures tested (CCI vs. sham F_1,392 _= 79., p < 10^-5^; SNI vs. sham F_1,392 _= 6.6, p < 0.01). Over the post-surgery test days (7, 17, 24), SNI vs. sham contrast was not significant at 40°C and 52°C, but was significant at 44°C (F_1,392 _= 4.8, p < 0.03) and 48°C (F_1,392 _= 47.5, p < 10^-5^). In comparison, CCI vs. sham contrasts, over post-surgery days, were significant for all temperatures tested (for 40°C F_1,392 _= 8.3, p < 0.004; for 44°C F_1,392 _= 6.3, p < 0.01; for 48°C F_1,392 _= 142., p < 10^-5^; for 52°C F_1,392 _= 5.6, p < 0.02).

### Operant dynamic heat sensitivity test – AlgoTrack – responses

**Figure 5:**
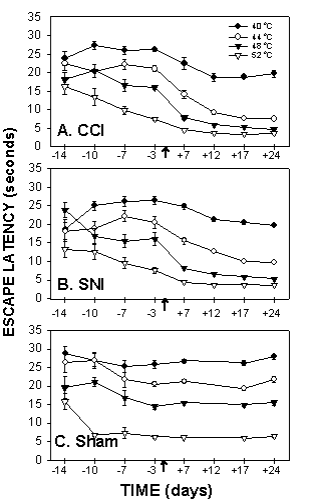
*AlgoTrack test for thermal sensitivity*, escape latencies, as a function of time in CCI (A), SNI (B) and sham (C) rats. The test was performed for all animals at 4 temperatures (40, 44, 48 and 52°C). Escape latencies were significantly reduced in CCI and SNI animals at all temperatures following ligation as compared to sham, and as compared to pre-ligation. The decrease in escape latencies was maximal at day 12 post-ligation and was maintained throughout the period of testing.

After appropriate training on this task (see below), all three animal groups (CCI, SNI, and sham) show a systematic decrease of escape latency with increasing temperatures applied to the floor (Fig. 5). Moreover, escape latencies for both SNI and CCI exhibited significant attenuation at all 4 temperatures after surgery as compared to pre-surgery values within each group and compared to sham (Fig. 5). 3-way ANOVA for escape latency as a function of animal grouping, day of test, and temperature tested was highly significant for all 3 factors (for animal grouping F_2,392 _= 107; for test day F_3,392 _= 76.62; and for test temperature F_3,392 _= 666.2; p < 10^-5 ^for all 3 factors), for all their pair-wise interactions (p < 10^-5^) and their 3 way interaction (p < 0.01). Contrasts between pre- and post surgery escape latencies were significant for CCI (F_1,392 _= 195, p < 10^-5^) and SNI (F_1,392 _= 148, p < 10^-5^) but not for sham (F_1,392 _= 0.96, p > .32) animals. Differences between pre- and post-surgery escape latencies in both SNI and CCI was highly significantly different at all 4 temperatures tested (F-tests, p < 10^-5^). The contrasts between CCI and sham, and between SNI and sham were significant across days and temperatures tested (CCI vs. sham F_1,392 _= 184, p < 10^-5^; SNI vs. sham F_1,392 _= 144, p < 10^-5^). Over the post-surgery test days (7, 17, 24), CCI vs. sham contrasts, over post-surgery days, were significant for all temperatures tested (for 40°C F_1,392 _= 53.7, p < 10^-5^; for 44°C F_1,392 _= 148.5, p < 10^-5^; for 48°C F_1,392 _= 105.7, p < 10^-5^; for 52°C F_1,392 _= 5.9, p < 0.01). Similarly, SNI vs. sham contrast was significant for all temperatures tested (for 40°C F_1,392 _= 35, p < 10^-5^; for 44°C F_1,392 _= 105, p < 10^-5^; for 48°C F_1,392 _= 99.7, p < 10^-5^; for 52°C F_1,392 _= 6.25, p < 0.01).

### Comparison between hotplate and AlgoTrack stimulus-response curves

**Figure 6:**
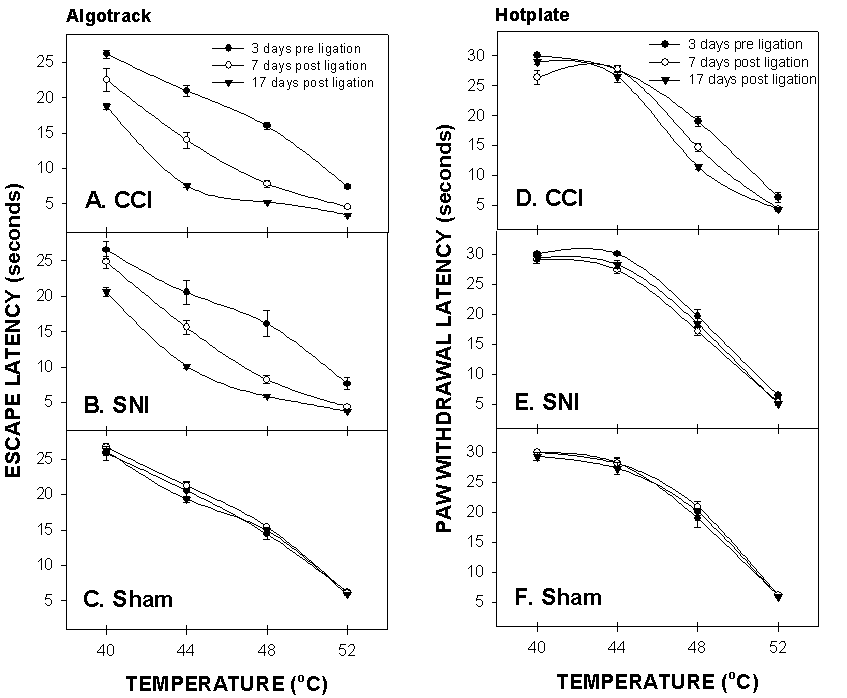
Comparison of the temperature-escape/paw withdrawal responses between AlgoTrack and Hotplate tests in CCI (A, D), SNI (B, E), and sham (C, F) animals tested at 3 days prior and at 7 and 17 days post-ligation. On the hotplate test, paw withdrawal latency decreases post-ligation are small and observed mainly in CCI rats. On the AlgoTrack test, both CCI (A) and SNI (B) rats exhibit post-ligation attenuation in their escape times at all temperatures tested.

Given that hotplate and AlgoTrack tests are the only supraspinal responses examined in this study, direct comparison between them is informative. CCI and SNI, but not sham, animals show similar leftward shifts in stimulus-response curves on both tests (Fig. 6). However, on the hotplate this leftward shift is only 2°C, while on AlgoTrack it is larger than 6°C.

### Comparing between Plantar, Hotplate and AlgoTrack for thermal hyperalgesia

**Figure 7:**
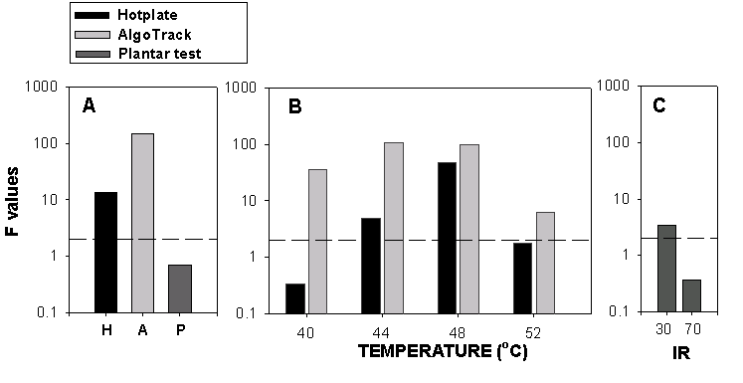
Comparing detection of thermal sensitivity changes between Plantar, Hotplate, and AlgoTrack tests in SNI rats (F-values in ANOVA planned comparisons). **A) **F-values for contrasting thermal pain behavior pre-ligation (3 days prior) to post-ligation (days 7, 17 and 24) on the Plantar test (P; for both 30 and 70 IR intensities); on the Hotplate test (H; for all test temperatures: 40, 44, 48, 52°C); and on the AlgoTrack test (for the same test temperatures). **B) **F-values for contrasting between SNI rats and sham rats on AlgoTrack test (gray bars) and Hotplate test (black bars) in post-ligation days (7, 17 and 24), for each applied temperature indicated. **C) **F-values for contrasting between SNI rats and sham rats on Plantar test in post-ligation days, for the IR intensities indicated. The y-axis is in log scale and covers 4 decades. The dashed line is for F = 2.0, which delineates threshold for significance.

Plantar, hotplate and AlgoTrack tests assess thermal pain behavior. To compare the outcomes on these tests, we use F-values obtained for contrasts examined in planned comparisons. Given that the largest differences between the three thermal tasks were seen in SNI animals, the F-values for pre- vs. post-ligation thermal responses, and for SNI vs. sham post-ligation thermal responses are summarized in figure 7. Behavioral outcomes on AlgoTrack show the largest thermal hyperalgesia, followed by outcomes on Hotplate, while outcomes on Plantar test show the least amount of thermal hyperalgesia.

### Performance on AlgoTrack is learned behavior

**Figure 8:**
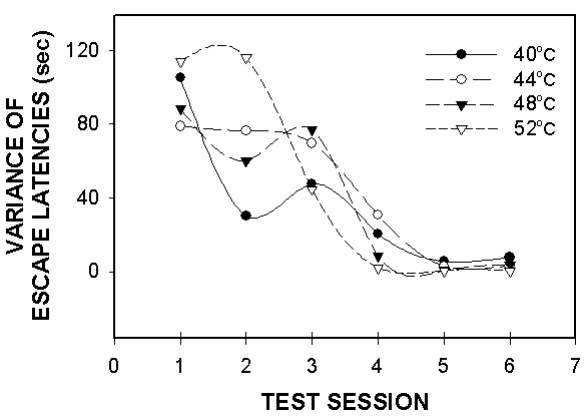
AlgoTrack escape latency variance in sham rats (n = 8) for 4 temperatures (40, 44, 48 and 52°C) as a function of test session. Variances for all temperatures tested decreased by the fourth testing session, and were maintained through further testing.

**Figure 9:**
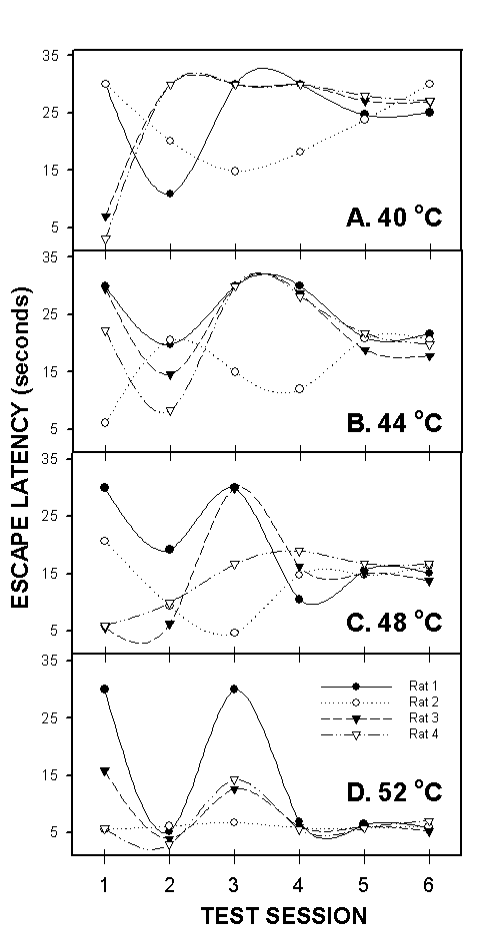
Algotrack individual responses of 4 sham rats for 4 temperatures 40 (A), 44 (B), 48 (C) and 52°C (D) plotted against test session. Illustrated are the first 6 sessions these animals were ever exposed to this paradigm. Each animal exhibits its own pattern of learning throughout the first few testing sessions, and then stabilizes to well-defined escape latencies for the different temperatures.

Unlike the hotplate and plantar tests, and because AlgoTrack involves more complex operant behavior, hence proper assessment of thermal responses on this device requires an initial period of training. Rats seem to need at least 4 testing sessions over a 2-week period to learn that escaping to the other plate eliminates the stimulus. These are seen in the initial escape values in figure 5. Figure 8 illustrates the effects of learning by showing that the variance of escape latencies in the sham rats decreased by two magnitudes over six test-sessions. Moreover, examining individual animal behavior shows that each animal undergoes a unique behavioral pattern of change and yet eventually settles to stereotyped escape behavior where escape latencies become predictable by the applied temperature and similar between animals (Fig. 9).

## Discussion

The main observation of the present result is that the presence and magnitude of thermal hyperalgesia, as assessed in two types of neuropathic rats, critically depends on the type of test used. Our automated, operant, dynamic thermal sensitivity assessment, AlgoTrack, seems most sensitive, hotplate test shows lower sensitivity, and the plantar test seems to be the least sensitive. We also show the ability to follow thermal stimulus-response curves in awake, freely moving rats, repeatedly for weeks, using our automated device.

### Comparison between pain assessment tools

A number of groups have attempted to develop automated behavior assessment tools to measure pain responses in animals in order to exclude bias from experimenters' judgments. Jett and Michelson [25] and Duysens and Gybles [26] developed a computer-driven force detector to measure animals' motor response accurately after painful stimuli. Jourdan et al. [27] introduced an automated pain scoring technique based on continuous video recording of movements of rats after formalin injection. Möller et al. [28] constructed an electronic pressure stimulator to standardize mechanical force applied to animals. The noxious heat stimulator first described by Hargreaves et al. [16], made a great impact on thermal pain behavior measurement due to several advantages: 1) animals are stimulated without restraining, thus minimizing stress and unnecessary sympathetic and hormonal response, 2) stimulus application and response measures are fully automated thereby minimizing human involvement in the assessment, and 3) the stimulus is applied to each limb separately. Although these advances have improved our understanding of animal pain behavior, these methods emphasize reflexive aspects of pain behavior. Recently LaBuda and Fuchs [29] described cognitive avoidance behavior test paradigm based on the combination of place preference and mechanical stimulation. Even though the paradigm does not eliminate human involvement, it does assess integrated behavior contrasting pain with aversion, and thus seems a very useful tool. Mauderli et al. [14] describe an alternative thermal pain assessment tool in awake, behaving rats. The paradigm is based on two boxes, one is used for thermal escape behavior, modulated by light/dark preference; while a second box provides a control task which measures escape latencies from bright light, controlling for generalized effects on aversion. Similarly to our device, the paradigm of Mauderli et al. [14] assesses pain behavior that depends on learned responses in the awake, behaving rat. Their paradigm also provides the means for dissociating the effects of analgesics on motor behavior from that of antinociception. The Mauderli et al. [14] and LaBuda and Fuchs [29] devices are complimentary to each other, contrasting mechanical and thermal pain with aversion. Yet outcomes from these devices have not been compared directly. Given the present results, we predict that outcomes on these devices may be different depending on type of injury giving rise to the pain behavior. We dub our device as a dynamic operant behavior task, to distinguish it from the paradigm of Mauderli et al. [14], since in our paradigm the animal responds to changes in skin temperature, while in the Mauderli paradigm the animal is actively exploring an environment where the floor temperature is kept constant.

Given that the our paradigm is dependent on learned behavior and on motor responses where the animal has to assess its body position relative to the track and find the appropriate escape path, implies that the responses are due to highly integrated behavior, which most likely depends on cortical circuitry. Obviously anesthetized or decorticated animals cannot do this task, which is not true for reflex-dependent pain assessment tools, e.g. the plantar or tail flick tests. Our device (AlgoTrack) is similar in design to a shuttle box introduced by Berkeley and Parmer [30] who used electrical shock applied through the floor for induction of pain and latency to escape over a raised barrier as a measure of pain behavior in awake, unrestrained, trained cats. Their study is important since it showed that lesions limited to the somatosensory cortex increased escape response thresholds. Hence demonstrating at least partial dependence of such behaviors on cortical circuits. The paradigm introduced by Berkeley and Parmer [30] has not been used in pain assessment since, perhaps because it was based on responses to electrical shock (electrical-shock induced escape behavior remains popular in studies of brain substrates underlying aversive classical conditional learning, e.g. LeDoux, [31], mainly due to the well-defined temporal relationship between conditioned and unconditioned stimuli), and perhaps due to the lack of convincing evidence at the time for involvement of the cerebral cortex in pain behavior/perception. Our device also shares similarities with both hotplate and plantar devices since it assesses escape times for thermal stimuli. Of course the plantar test is based on local monosynaptic reflex circuitry, while hotplate and AlgoTrack involve more complex behavior. It should be emphasized that the monitoring of local sign in the plantar test is a main advantage since the measure can distinguish thermal sensitivity differences between injured and uninjured limbs. The AlgoTrack sacrifices this differentiation to assess the significance of thermal stimuli to the whole organism, in a completely automated fashion. It is based on the assumption that the escape behavior is triggered primarily through tissue that is most sensitive to the stimulus, in this case the injured limb. It should be mentioned that AlgoTrack is rather labor intensive as compared to simple reflexive tests, making it difficult to implement in high throughput studies. It also requires ability to learn and this may become a confounder in genetic manipulations where learning may be impaired.

### Caveats regarding learning and over-learning

Our results show that AlgoTrack requires an initial period of training of about 4 sessions, over a 2-week interval. In the first training trials, escape behavior exhibits large variability because the animals need to discover that moving to the opposite side would eliminate the stimulus, and often exhibit agitated behavior including licking, jumping, as well as exploring. Moreover, in the initial trials many animals exhibit side preference. After training the overall variance decreased, and the mean escape duration was very similar across different animals. This dependence on learning indicates involvement of cortical circuitry.

Various precautions were implemented to limit or decrease over-learned behavior: 1. Non-noxious, or minimally noxious, thermal stimuli (40 and 44°C) were inter-mixed with more noxious stimuli (>45°C), to reduce stimulus predictability. 2. Thermal stimuli were presented in a pseudo-random sequence. 3. Behavioral assessment was limited to two test sessions per week. The results indicate that we have overcome these difficulties since in the sham-operated rats there is a relatively constant stimulus-response curve for the duration of the study (38 days), past the initial period of training. Not presented are observations we have made in other animals where more frequent testing on the AlgoTrack (> 4 tests per week) resulted in further decreases in escape latencies and decreased sensitivity to stimulus temperature variations.

### Neuropathic pain behavior and differential thermal hyperalgesia

Overall the quantitative results assessing pain-like behavior shown for CCI and SNI rats closely agree to earlier results, demonstrating that both groups show increased mechanical sensitivity as determined by decreased thresholds to von Frey filaments. SNI animals also exhibited cold sensitivity as measured by the acetone drop test [24,32,33]. CCI animals showed thermal hyperalgesia as measured by the plantar and hotplate test [23,34-36]. The new result is the illustration that both groups exhibit more profound thermal hyperalgesia than suspected in the past when they are tested on our automated paradigm (AlgoTrack). The F-values for comparing pain behavior in SNI animals pre- to post-ligation indicates a 10-fold increase in detectability of hyperalgesia by AlgoTrack as compared to plantar or hotplate tests. Moreover, detectability of hyperalgesia by AlgoTrack of SNI rats as compared to sham post-ligation also show consistently higher F-values than on hotplate or plantar tests.

Multiple reasons may underlie the observed thermal sensitivity differences. The differences may be a reflection of the specifics of executing the different tests, and/or a reflection of the animal models studied. Regarding the details of the tests: Since our paradigm is completely automated its results must be considered the most reliable. On the other hand, the plantar test is very simple to deliver and the outcome measure is automated. It is possible that the application of the heat during the plantar test is more compromised because of the abnormal paw position that SNI animals exhibit, and because of the large tactile sensitivity differences these animals show between lateral and medial aspects of the paw. Alternatively, or in addition the thermal sensitivity differences may reflect the level of the central nervous system circuitry assessed by each test and its significance to the pain model understudy. The existence of multiple distinct central nociceptive circuits has been suggested in the past [19,37-39]. We presume that the more complex behavioral requirement translates into engaging higher central circuitry; as a result the plantar test reflects local reflexes, the hotplate bulbo-spinal pathways, while the AlgoTrack may require cortical nociceptive circuitry. Therefore, our results can be interpreted as suggesting that, in neuropathic injury rats, thermal hyperalgesia becomes more prominent when the task requires more integrated behavior. This in turn implies that 'central sensitization' in these models more prominently includes supraspinal nociceptive circuitry. Based on this interpretation, we predict that pain models impinging more directly on peripheral processes, such as inflammatory conditions, might exhibit a test-dependence pattern of thermal hyperalgesia opposite in sensitivity to that observed for neuropathic animals. These hypotheses remain to be tested.

## Methods

### Hardware for automated thermal sensitivity testing

The device consists of a small rectangular box placed upon a metallic floor with 2 separate heating plates. The heating elements are made with flexible etched foil resistance, and covered with self-adhesive aluminum foil that maximizes thermal conductance to the plates. Plate temperatures increase at a rate of 5°C per second and are controlled by microprocessor-based temperature controllers (Odgen, Chicago, IL.). Initially both plates are set to a warm, comfortable temperature (36°C). A motion detector based on an array of infrared sensors, identifies on which of the two plates the animal is located. This plate is heated to a desired temperature. Upon perceiving the thermal stimulus, the animal typically escapes to the opposite non-heated plate. The infrared sensors detect the crossing of the animal and both escape latency and temperature are displayed. The controller switches the heated plate to the baseline temperature and, the whole operation is repeated after a 5-minute inter-trial interval.

### Animals

Twenty-six male Sprague-Dawley rats (250–350 grams) were used throughout this study. They were maintained in a climate-controlled environment on a 12-hour light/dark cycle with free access to food and water. All tests were performed in the light period and were approved by the Animal Care and Use Committee (ACUC) at Northwestern University, Chicago, and were in accordance with the NIH for the ethical use of laboratory animals and the IASP for use of conscious animals in pain research [40].

### Surgery

Animals were anesthetized using ketamine hydrochloride (45 mg/kg, i.p.) and xylazine (10 mg/kg i.p.) After surgery, all wounds were sutured using a non-absorbable surgical suture (3-0 silk, REF 1124-11, Sherwood Medical, USA), treated with a topical antibiotic ointment, and were rested for seven days before further testing. The SNI model was implemented in 10 animals. The left sciatic nerve was exposed at the level of its trifurcation into the sural, tibial and common peroneal nerves. Each of the tibial and common peroneal nerve was tightly ligated by two knots 4 mm apart using 6.0 silk and then completely severed in between, leaving the sural nerve intact [24]. The CCI model was implemented in a second group of animals (n = 8). In these animals, the left sciatic nerve was exposed above the level of trifurcation and four loose knots were carefully applied to the nerve using absorbable gut [23]. An additional 8 animals were used as sham, where the sciatic nerve was exposed but not manipulated.

### Pain behavioral testing

All operated animals (CCI, SNI and sham rats) were studied behaviorally from 2 weeks prior to surgery up to 4 weeks post surgery. Mechanical and cold sensitivities as well as thermal responses on AlgoTrack, Hotplate, and Plantar test were examined in all animals, at all time points.

### Mechanical sensitivity

Mechanical sensitivity of the hind paw was measured by determining withdrawal thresholds to Von Frey filaments. A set of 18 filaments (Stoelting, Chicago, IL.), marked from 1.65 to 6.5, was used. The respective bending forces were in the range of 0.005 to 125.892 g. The animals were placed individually in a small (35 × 20 × 15 cm) plastic cage with an open wire mesh bottom. Before testing, the rats were left in the test cages for 15–20 min so that their grooming and exploratory behaviors cease and all four paws were placed on the ground. All tests were performed on the right (control) and left (ligated) hind paws. Von Frey filaments were applied perpendicularly to the planter surface of the paw with an upward force just sufficient to bend the microfilament. When testing SNI animals, special care was taken to stimulate the lateral plantar surface, which is the area of the skin innervated by the sural nerve [24]. Paw withdrawals due to locomotion or weight shifting were not counted and such trials were repeated. The 50% threshold for each paw withdrawal was calculated as described by Chaplan et al. [13].

### Cold sensitivity

Cold allodynia was measured by the acetone drop test described by Décosterd and Woolf [24]. A blunt needle connected to a syringe was used to drop 50 μl of acetone on the paw and the duration (in seconds) of the paw withdrawal was recorded. Minimal and maximal cut-offs were assigned at 0.5 and 20 sec, respectively. Paw withdrawals due to locomotion or weight shifting were not counted and such trials were repeated.

### Plantar test

Animals were placed in an acrylic box with glass pane floor and the plantar surface of their hind paw was exposed to a beam of infrared radiant heat (Ugo Basile, Stoelting, Chicago, IL; [16]). The paw withdrawal latencies were recorded at 2 different infrared intensities, 30 and 70, and were measured twice per session, separated by a minimum interval of 5 minutes. Minimum and maximum cut-offs were assigned at 1 and 30 seconds, respectively. Again, paw withdrawals due to locomotion or weight shifting were not counted and the trials repeated.

### Hotplate test

Animals were gently dropped into an acrylic box with a metal floor that was preheated to a certain temperature [17]. Paw withdrawal latency was calculated using a timer that was started when the animal is released onto the preheated plate and stopped at the moment of withdrawal, shaking, or licking of either hind paw. Paw withdrawal latencies for each animal were calculated for 4 different temperatures (40, 44, 48, and 52°C). All animals were tested once for each temperature per session in a random sequence.

### Automated dynamic thermal sensitivity test (AlgoTrack)

Animals were gently dropped into the chamber with both plates preheated to 36°C and allowed to rest for 5 minutes. Then the plate on which the rat is rested is heated up to the desired temperature. Escape latencies were recorded for all the animals at 4 different temperatures (40, 44, 48, and 52°C). Animals were tested twice for each temperature per session, with the temperatures presented in a randomized sequence. The maximum thermal stimulus duration was 30 seconds. If an animal did not escape a stimulus in this time duration, the stimulus was turned off and the animal's response recorded as escape at 30 seconds. Animals tested on this paradigm did not exhibit any signs of tissue injury or burns at all temperatures tested, throughout the period of the study.

### Data analysis

All data are presented as group averages ± SEM. The baseline value for all tests between pre- and post neuropathic injury used the last test session prior to operation (day -3). The differences between animal groupings (CCI, SNI, sham), day of test (-3, 7, 17, and 24 days), ligated and non-ligated hind-paws (left and right), and different intensities of stimuli were compared with multi-way between-groups fixed-effects analysis of variance (ANOVA). Planned comparisons were tested using contrast analysis (F-tests), and post-hoc comparisons were done using Turkey's honest test for post-hoc multiple comparisons for unequal N. Statistical significance was considered at p < 0.05 (Statistica, StatSoft, Inc.).

## Competing interests

The author(s) declare that they have no competing interests.
